# Efficacy of THN201, a Combination of Donepezil and Mefloquine, to Reverse Neurocognitive Deficits in Alzheimer’s Disease

**DOI:** 10.3389/fnins.2020.00563

**Published:** 2020-06-16

**Authors:** Marine Droguerre, Adeline Duchêne, Christèle Picoli, Benjamin Portal, Camille Lejards, Bruno P. Guiard, Johann Meunier, Vanessa Villard, Nicole Déglon, Michel Hamon, Franck Mouthon, Mathieu Charvériat

**Affiliations:** ^1^Theranexus, Lyon, France; ^2^Centre de Recherches sur la Cognition Animale (CRCA), Centre de Biologie Intégrative (CBI), CNRS, UPS, Université de Toulouse, Toulouse, France; ^3^Amylgen, Montferrier-sur-Lez, France; ^4^Laboratory of Neurotherapies and NeuroModulation, Neuroscience Research Center (CRN), University of Lausanne, Lausanne, Switzerland; ^5^Laboratory of Neurotherapies and NeuroModulation, Department of Clinical Neuroscience (DNC), Lausanne University Hospital (CHUV), Lausanne, Switzerland

**Keywords:** Alzheimer’s disease, donepezil, mefloquine, connexin, astroglial networks, amyloid beta, behavioral tests

## Abstract

Donepezil (DPZ) is an acetylcholinesterase inhibitor used in Alzheimer’s disease to restore cognitive functions but is endowed with limited efficacy. Recent studies pointed out the implication of astroglial networks in cognitive processes, notably via astrocyte connexins (Cxs), proteins involved in gap junction intercellular communications. Hence, we investigated the impact on cognition of pharmacological or genetic modulations of those astrocyte Cxs during DPZ challenge in two rodent models of Alzheimer’s disease–like memory deficits. We demonstrated that the Cx modulator mefloquine (MEF) significantly enhanced the procognitive effect of DPZ in both models. In parallel, we determined that MEF potentiated DPZ-induced release of acetylcholine in hippocampus. Finally, local genetic silencing of astrocyte Cxs in the hippocampus was also found to enhance the procognitive effect of DPZ, pointing out the importance of Cx-dependent astrocyte networks in memory processes.

## Introduction

Alzheimer’s disease (AD) is characterized by the loss of cognitive functions especially learning, working, and spatial memory (Mattila et al., 2012). Brains from AD patients are notably distinguished by senile plaques composed of amyloid β (Aβ) protein and a marked deficit in acetylcholine (ACh) notably in the hippocampus (Shinotoh et al., 2000). Donepezil (DPZ), a potent acetylcholinesterase (AChE) inhibitor, is currently one of the most widely used for AD in the world (Adlimoghaddam et al., 2018). Through its reversible binding to AChE, DPZ promotes cholinergic neurotransmission, thereby alleviating cognitive impairments. However, only modest improvements were found in patients, and the development of novel therapeutic approaches is eagerly needed (Deardorff et al., 2015).

Numerous studies have shown that astrocytes modulate neuronal activity (Dallerac and Rouach, 2016; Charvériat et al., 2017). These glial cells highly express gap junction proteins (Bennett et al., 2003), named connexins (Cxs), responsible for the organization of astrocytes into dynamically networks, which play key roles in the regulation of brain functions (Han et al., 2014; Dallerac and Rouach, 2016; Clasadonte et al., 2017). Connexin 30 and Cx43 are principal constituents of astroglial gap junctions (Nagy and Rash, 2000). As Cxs are dysregulated in AD, it has been suggested that they might be relevant targets to improve cognitive performances (Giaume et al., 2017). In this study, we postulate that Cx-based astroglial networks might modulate DPZ pharmacological effects, and use validated mouse models for assessing our hypothesis. Mefloquine (MEF) was selected as a pharmacological tool to modulate astroglial Cxs, as previously reported both *in vitro* and *in vivo* (Picoli et al., 2012, 2019; Jeanson et al., 2016; Droguerre et al., 2019).

The pharmacological profile of DPZ alone or combined with MEF at low dose (combination THN201) was investigated on cognitive performances in scopolamine and Aβ mouse models of AD to assess learning, working, and spatial memories. In parallel, pharmacokinetic and pharmacodynamic interactions of DPZ and MEF were investigated using bioanalytical determinations of both drugs in serum and brain and *in vivo* microdialysis at hippocampal level. Finally, the implication of astroglial Cxs was further investigated using recombinant lentiviruses to specifically silence hippocampal expression of astroglial Cx30 and Cx43. To our knowledge, this study is the first to address the role of astrocyte Cxs as new therapeutic targets to enhance DPZ efficacy and provides new insights in the importance of glial cells in memory function.

## Materials and Methods

### Animals

All experiments were conducted in strict conformity with the Policies of the French Ethics Committee. Animal surgery and experimentations conducted in this study were authorized by the French Direction of Veterinary Services (APAFIS#12311-2017071816217821 v3). Swiss and C57BL/6 male mice (age 5 weeks; 30–35 g) were purchased from Janvier Labs (Le Genest-Saint-Isle, France) and Envigo (Gannat, France), respectively. Sprague–Dawley (8-week-old) male rats were purchased from Janvier Labs. During all of the experimental period, rodents were group-housed (six per cage) and maintained under controlled environmental conditions (12-h light/12-h dark cycle; temperature of 23°C ± 2°C; humidity of 50 ± 10%) with food and water *ad libitum.*

### Drugs and Administration Procedures

#### Acute Treatments in Mice

Mefloquine (Sigma-Aldrich, L’Isle d’Abeau Chesnes, France) and DPZ (Sigma-Aldrich) were dissolved in 2% dimethyl sulfoxide (DMSO) and administered acutely either intraperitoneally (i.p.) (5 mL/kg) or by oral gavage (p.o.) (10 mL/kg). Scopolamine (Sigma-Aldrich) was administered subcutaneously (s.c.) (5 mL/kg) in saline solution (0.9% NaCl).

#### Repeated Treatments in Mice and Rats

For daily administration for 14 days, animals received the drugs at various doses p.o. (see section “Results”) in a volume of 10 mL/kg. Control groups received the vehicles only under the very same conditions (volume, time, route of administration) as tested drugs.

#### Intracerebroventricular Injection of Aβ_25__–__35_

Both peptides Aβ_25__–__35_ and Sc.Aβ (scramble Aβ_25__–__35_) were purchased from PolyPeptides (Strasbourg, France). Swiss male mice were anesthetized with isoflurane 2.5% and administered intracerebroventricularly (i.c.v.) with 9 nmol of Aβ_25__–__35_ peptide or Sc.Aβ peptide in a volume of 3 μL per mouse, as previously described (Maurice et al., 1996; Villard et al., 2011b).

#### Stereotaxic Injection of Lentiviral Vectors

MOKOLA pseudotyped lentiviral vectors (LVs) encoding an shRNA directed against either Cx30, Cx43, or GFP (control) mRNA within astrocytes were generated as previously described (Colin et al., 2009; Quesseveur et al., 2015). The viruses were suspended in phosphate-buffered saline (PBS, 0.1 M, pH 7.4) containing 1% bovine serum albumin (BSA) to reach a final concentration of 150,000 ng/p24 for sh-Cx30 and 100,000 ng/p24 for both sh-Cx43 and sh-GFP. Mice were anesthetized with ketamine 75 mg/kg i.p., (Sigma-Aldrich) and xylazine 10 mg/kg i.p., (Sigma-Aldrich) and received two intrahippocampal injections (one per side) of either LV suspension in a total volume of 1 μL per injection, at a rate of 0.1 μL/min. The stereotaxic coordinates for the bilateral injections into the hippocampus were anteroposterior: −2.2, lateral: ±2.5, and ventral: −2.5 (in mm from bregma, according to Franklin and Paxinos (2007). After completion of the injection, the needle was left in place for 5 min before being slowly removed. Animals were allowed to rest under a warming lamp until full recovery from anesthesia and then returned back to their home cage under standard environmental conditions.

### Memory Behavioral Testing

#### Y-Maze

Mice were placed at the end of one arm of a Y-maze and allowed to move freely through the maze during an 8-min session. Spontaneous alternation was defined as entries into all three arms on consecutive occasions. The percentage alternation was calculated as the ratio of the [number of alternations] over the [total arm entries −2] × 100. The number of total arm entries during the 8-min session was used to quantify locomotor activity. Animals showing an extreme alternation percentage (<20 or >90%) were discarded from the analysis (Villard et al., 2011a).

#### Object Recognition/Location Test

Both novel object recognition (NOR) and novel object location (NOL) tests were carried out in an open-field arena during three phases (Leger et al., 2013; Vogel-Ciernia and Wood, 2014; Lueptow, 2017). (i) For the habituation phase on the first day, mice were allowed to freely explore the empty apparatus. (ii) On the second day, two identical objects were placed at two opposite edges of the arena. Time spent by the mouse exploring the two objects was recorded. (iii) Twenty-four hours later, one of the two familiar objects was replaced by a novel one (NOR) or displaced (NOL). Mice were allowed to explore the whole arena during 10 min for each of the three phases. The preference index was calculated as the ratio of the duration of contacts with the novel/displaced object over the total duration of contacts with the two objects. Locomotion was recorded using EthoVision software (Noldus, Paris, France).

#### Morris Water Maze

Mice were placed in a circular pool with external cues in the room. A transparent platform was immersed under the water surface during learning phases (Konsolaki et al., 2016). Training consisted of three swims with 20-min intertrial time each day for 5 days, performed between days 11 and 15 after Aβ_25__–__35_ peptide or Sc.Aβ peptide i.c.v. injection. Random starting positions were selected each day, and each animal was allowed a 90-s swim to find the platform. Probe test was performed 24 h after the last swim (day 16 after peptide injection). The platform was removed, and each animal was allowed a free 60-s swim. The time spent in each quadrant was determined. Swimming was recorded using EthoVision software (Noldus).

### Microdialysis Procedure and Quantification of ACh

Mice were anesthetized with isoflurane, placed in a stereotaxic frame and bilaterally implanted with probes in both left and right hippocampi. The stereotaxic coordinates for the bilateral injections into the hippocampus were anteroposterior: −2.4, lateral: ±2.7, and ventral: −3 (in mm from bregma, according to Franklin and Paxinos (2007). Microdialysis experiments were conducted with artificial cerebrospinal fluid (aCSF) in freely moving mice 24 h after surgery as previously described (Guiard et al., 2005). Dialysate samples were collected each 20 min and analyzed for ACh contents using an ultrahigh-performance liquid chromatography (UHPLC) method (see below). Basal values were determined for each mouse, and changes in ACh outflow induced by acute administration of vehicle, DPZ alone, or in combination with MEF were expressed as percentage of these basal values.

Concentrations of ACh in the microdialysis samples were analyzed by the Pronexus Analytical AB company (Stockholm, Sweden) using UHPLC tandem mass spectrometry (MS/MS). Briefly, the UHPLC-MS/MS system included a PAL autosampler (CTC Analytics, Zwingen, Switzerland), an Advance UHPLC pump, and an EVOQ Elite triple quadrupole mass spectrometer (Bruker Daltonics, Fremont, CA, United States) equipped with electrospray ionization source operating in a positive mode at +4,500 V. The source parameters were as follows: probe gas flow: 50, nebulizer gas flow: 60, probe temperature: +300°C, cone gas flow: 30, cone temperature: +200°C, CID gas: Ar 1.5 mTorr. A Titan PFP column (100 × 2.1 mm, 1.9 μm, 120 Å pore size) purchased from Sigma-Aldrich (Sweden) was used for UHPLC. Deuterated ACh (acetylcholine-1,1,2,2-d4 bromide; ACh-d4), used as internal standard, was purchased from C/D/N Isotopes (QMX Laboratories, Dunmow CM6 2PY, United Kingdom). Typically, 10 μL of aCSF or hippocampal microdialysate was mixed with 20 μL of ACh-d4 internal standard (50 nM, in aCSF), and 5 μL of the mixture was injected into the column. The mobile phase A was 0.1% (vol/vol) formic acid in water; the mobile phase B was acetonitrile with 0.1% formic acid. The linear elution gradient was as follows: 0–0.5 min: 5% B; 3.5 min: 55% B; 3.6 to 4.0 min: 70% B; 4.1 min, 5% B. The flow rate was 400 μL/min, and the total run-to-run time was 4.8 min. The calibration curve was linear in the range of 0.1 to 409.6 nM ACh (*R*^2^ = 0.999); the estimated limit of detection was 0.05 nM, and the lower limit of quantification was 0.15 nM.

### AChE Activity

Acetylcholinesterase activity was quantified using a cholinesterase activity assay kit according to manufacturer’s recommendations (MAK119; Sigma, St. Louis, MO, United States). Briefly, mouse hippocampus was homogenized (10 μL/mg) in 0.1 M Na/K phosphate buffer (PB) (pH 7.5) at 4°C followed by centrifugation at 30,000 × *g* for 5 min. Aliquots of the cleared supernatants were added to the kit reaction mixture containing various concentrations of DPZ (1–50 nM) and/or MEF (0.5–4 μM), and the reaction proceeded at room temperature. Absorbance at 415 nm was quantified using VICTOR plate reader (Perkin–Elmer Inc., Waltham, Massachusetts, United States) at 2-min intervals from time 0 (tissue sample addition) up to 10 min. Acetylcholinesterase activity was expressed as units/L. One unit of AChE is the amount of enzyme that catalyzes the production of 1.0 μmol of thiocholine per minute at room temperature at pH 7.5.

### Pharmacokinetics of DPZ and MEF in Serum and Brain

These experiments had to be performed in rats because blood volume in mice was too low to allow collection of serial serum samples for studies over 24 h after drug administration.

#### Experimental Procedure

Rats were randomly assigned to one of three experimental groups: (i) DPZ 0.25 mg/kg; (ii) THN201: DPZ 0.25 mg/kg + MEF 1 mg/kg; (iii) DPZ 1 mg/kg per day. Drugs were administered p.o., once per day for 14 days.

#### Tissue Preparation

Blood was collected on the 14th treatment day, just prior to drug administration (T0) and then 30 min, 1, 2, 4, 8, and 24 h later. Blood was collected by retro-orbital puncture except for the last collection, which was made by intracardiac puncture. After coagulation, blood samples were centrifuged at 3,000 × *g* for 15 min, and serum was collected and stored at −80°C. Rats were sacrificed by decapitation immediately after the last blood collection, and their brains including brainstem and cerebellum were removed as one piece, placed on tinfoil precooled on dry ice and stored at −80°C until drug quantifications.

#### DPZ and MEF Quantification

One volume of brain or serum samples was added to three volumes of human serum EDTA K3, and the mixture was homogenized under magnetic stirring for 15 min for analysis using a Sciex API4000 Qtrap mass spectrometer coupled to a Shimadzu HPLC system (Shimadzu Corporation, Marne-la-Vallée, France). Liquid chromatography–MS procedures yielded detection ranges for DPZ from 0.1 to 100 ng/mL in serum and 0.1 to 100 ng/g in brain, and for MEF from 0.5 to 500 ng/mL in serum and 0.5 to 500 ng/g in brain.

### Immunohistochemistry and Quantification of Cx30 and Cx43 Expression

Mice were deeply anesthetized and transcardially perfused with a 4% paraformaldehyde solution in Na/K PB (0.4 M, pH 7.4). Brains were removed, and series of one in twelve 30-μm-thick coronal sections were cut at hippocampal level, rinsed in PBS, and then incubated 1 h in a blocking solution containing 10% of normal donkey serum (Bio-Rad, Marnes-la-Coquette, France) and 0.1% BSA (Euromedex, Souffelweyersheim, France) in PBS supplemented with 0.25% Triton-X100 (PBST). Primary antibodies were goat anti-GFAP (1:500, Abcam, Paris, France), mouse anti-Cx43 (1:250, BD transduction, Le Pont de Claix, France), and rabbit anti-Cx30 (1:250, ThermoScientific, Les Ulis, France) and diluted in blocking solution and applied overnight at 4°C. Sections at the very level of injection sites and displaying signs of lesions were discarded. After several rinses in PBST, sections were incubated for 120 min at room temperature in a mixture of Alexa Fluor^®^ 488–conjugated highly cross-adsorbed donkey anti-rabbit, Alexa Fluor^®^ 555–conjugated donkey anti-mouse, and Alexa Fluor^®^ 647–conjugated donkey anti-goat antibodies (all at 1:500; Life Technologies, ThermoScientific) in blocking solution. Sections were then rinsed extensively and finally mounted onto glass slides, cover slipped using Mowiol^®^ + Hoechst (1:10,000), and stored at 4°C.

Immunolabeled hippocampi from LV-injected mice were pictured using an Olympus BX-51 microscope equipped with Mercator Software (Explora Nova, La Rochelle, France). The area of interest was framed at ×20 magnification lens for each injection site and visualized on DAPI-labeled sections. Quantifications of DAPI, GFAP, Cx30, and Cx43 were conducted with the FIJI software (ImageJ 2; National Institutes of Health, Bethesda, MD, United States). Gray values for the GFAP, Cx30, and Cx43 immunolabeling in the area of interest were determined in each series of hippocampus sections.

### Statistical Analysis

All data are expressed as mean ± SEM, and normality of data distribution was evaluated using the Shapiro–Wilk normality test. Alternation performance and object exploration time were converted to percentage of alternation or preference index, respectively. Spontaneous alternation results were analyzed by two-way ANOVA followed by Tukey multiple-comparisons test. Novel object recognition or location results were analyzed using one-sample *t*-test vs chance level. Morris water maze (MWM) task was analyzed using two-way repeated-measures ANOVA followed by Tukey multiple-comparisons test (place-learning) and one-sample *t*-test vs chance level (probe test). Hippocampal ACh outflow was analyzed by two-way ANOVA followed by Fisher least significant difference *post hoc* test. Serum and brain levels of DPZ and MEF were analyzed by two-way ANOVA followed by Bonferroni *post hoc* test and Kruskal–Wallis test followed by Dunn *post hoc* test, respectively. Connexin 30 and Cx43 expressions were analyzed by two-way ANOVA followed by Tukey multiple-comparisons test. Significance level was set at 0.05.

## Results

### Mefloquine Potentiated the Effects of DPZ on Working Memory After a Scopolamine Challenge

**FIGURE 1:**
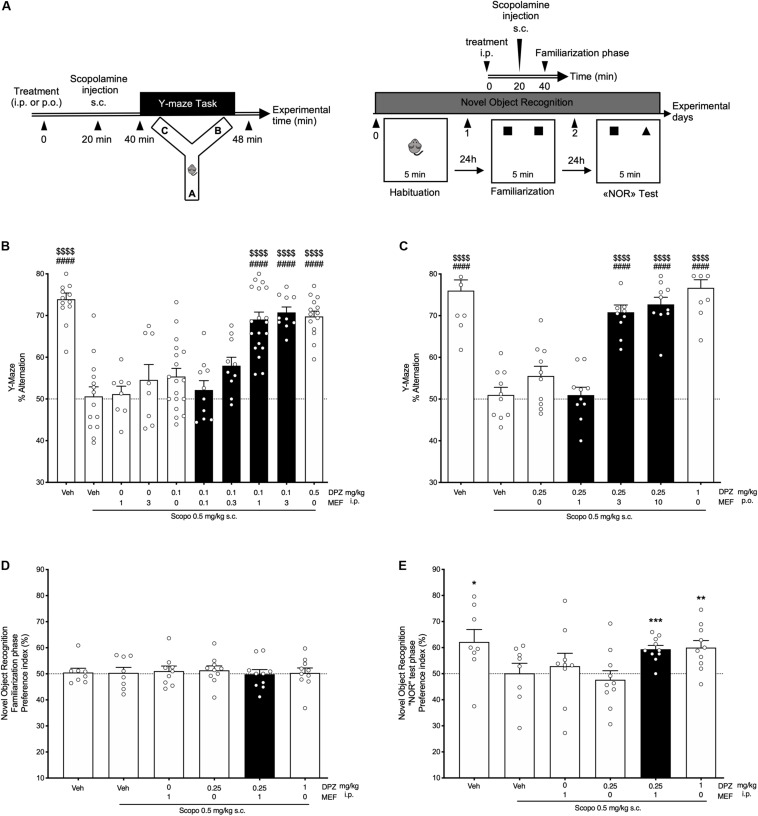
Behavioral performances in scopolamine-treated mice. Effects of donepezil and/or mefloquine (MEF). **(A)** Experimental design. **(B,C)** Spontaneous alternation task. **(B)** Mefloquine (0.1, 0.3, 1, 3 mg/kg), donepezil (DPZ, 0.1, 0.5 mg/kg), or vehicle (veh, DMSO 2%, NaCl 0.9%) was administered i.p., 40 min before the task. **(C)** Mefloquine (1, 3, 10 mg/kg), DPZ (0.25, 1 mg/kg), or veh (DMSO 2%) was administered p.o., 40 min before the task. **(B,C)** Scopolamine (scopo, 0.5 mg/kg) was administered s.c. 20 min after each treatment, that is, 20 min before the task. One-way ANOVA followed by Tukey multiple-comparisons test: ^####^*P* < 0.0001 vs scopo/veh, ^$$$$^*P* < 0.0001 vs scopo/DPZ0.1 **(B)** and vs scopo/DPZ0.25 **(C)**. **(D,E)** Novel object recognition task. Mefloquine (1 mg/kg), DPZ (0.25 or 1 mg/kg), or veh (DMSO 2%, NaCl 0.9%) was administered i.p., 40 min before the familiarization phase. Scopolamine (scopo, 0.5 mg/kg, s.c.) was administered 20 min after each treatment, that is, 20 min before the task. Preference index was determined (see section “Materials and Methods”) for the familiarization phase **(D)** and the novel object recognition test phase **(E)**. One-sample *t*-test: *P < 0.05; **P < 0.01; ***P < 0.001; vs chance level. **(B–E)** Bars in black indicate combination treatments. Data are expressed as mean ± SEM, *n* = 8 to 20 mice per group. Each experiment was performed using different cohorts.

Different cohorts of mice were tested using the Y-maze alternation task after i.p., or p.o., administration of DPZ either alone or in combination with MEF (Figure 1A, left panel). Regardless of the route of administration, scopolamine significantly impaired spontaneous alternation compared to vehicle-treated mice (*P* < 0.0001; Figures 1B,C). Whereas DPZ at 0.1 mg/kg (DPZ0.1) i.p., exerted no effect (*P* = 0.69; Figure 1B), DPZ 0.5 mg/kg (DPZ0.5) significantly reversed the scopolamine-induced deficit (*P* < 0.0001; Figure 1B). DPZ0.1, when combined with MEF at 1 or 3 mg/kg (DPZ0.1 + MEF1 and DPZ0.1 + MEF3), restored spontaneous alternation up to the control group (Figure 1B). Similar observations were made in mice that received treatment of DPZ0.25 p.o., which did not reverse scopolamine-induced deficit (*P* = 0.69; Figure 1C), but the same low dose of DPZ combined with MEF at 3 or 10 mg/kg p.o., significantly reversed the alternation deficit (both at *P* < 0.0001; Figure 1C), up to that noted in vehicle-treated controls, suggesting a potential synergistic effect of MEF and DPZ on working memory after a scopolamine challenge.

When subjected to the NOR task (Figure 1A, right panel), all mice spent the same amount of time exploring both identical objects during the familiarization session (all, *P* > 0.05; Figure 1D). In contrast, during the NOR test session, scopolamine administration abolished the increase in exploration time of the novel object observed in control group. Neither MEF1 i.p., nor DPZ0.25 i.p., prevented the scopolamine effect (*P* = 0.57 and *P* = 0.52, Figure 1E). However, the combination of drugs (DPZ0.25 + MEF1) was as efficient as DPZ at 1 mg/kg to restore exploration preference of the novel object in scopolamine-treated mice (Figure 1E).

### Mefloquine Potentiated the Effects of DPZ on Working Memory in a Mouse AD Model

**FIGURE 2:**
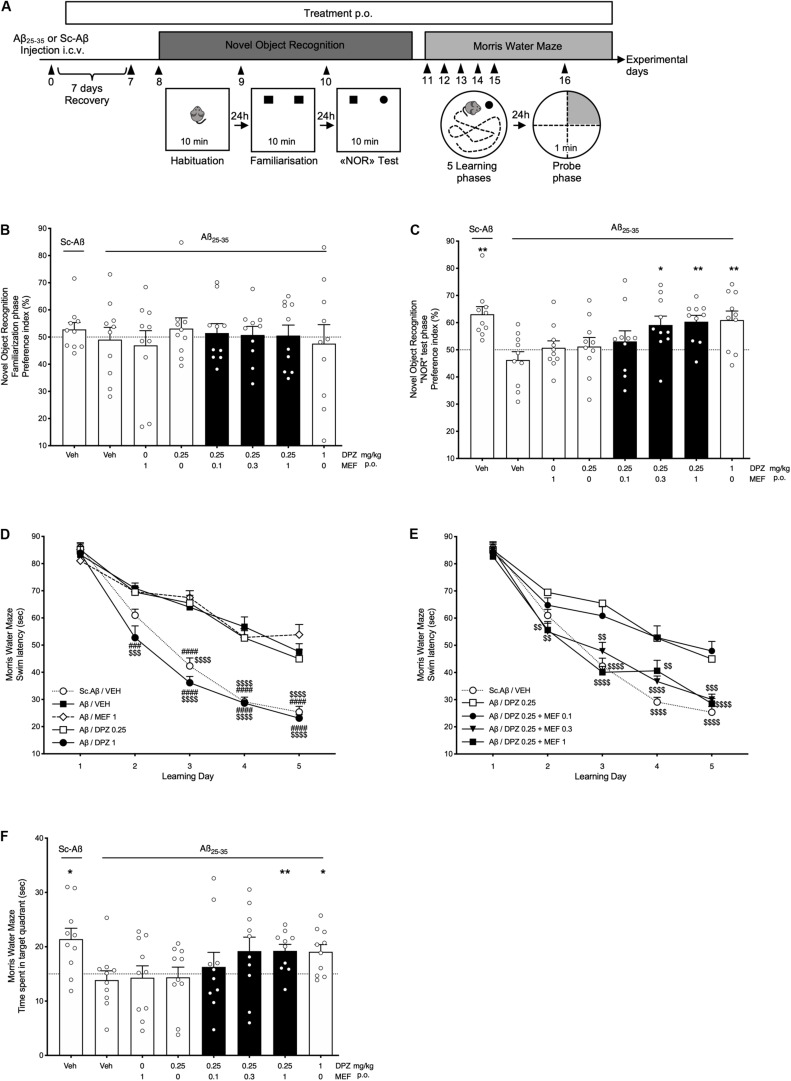
Behavioral performances in Aβ_25__–__35_ or scramble-Aβ peptide-injected mice. Effects of donepezil and/or mefloquine (MEF). **(A)** Experimental design. **(B–F)** Mefloquine (0.1, 0.3, 1 mg/kg), donepezil (DPZ, 0.25, 1 mg/kg), or vehicle (veh, DMSO 2%) was administered p.o. daily, from the day after i.c.v. injection (day 1) of Aβ_25__–__35_ peptide (Aβ) or scramble-Aβ peptide (Sc.Aβ) until the last day of experiment (day 16). From days 8 to 10, the preference index for identical objects **(B)** and novel object **(C)** in the open field was recorded for 10 min. One-sample *t*-test: ^∗^*P* < 0.05 and ^∗∗^*P* < 0.01 vs chance level. **(D,E)** Swim latency to reach an immerged platform was assessed in the Morris water maze test from days 11 to 15. Two-way repeated-measures ANOVA followed by Tukey multiple-comparisons test: ^###^*P* < 0.001, ^####^*P* < 0.0001 vs Aβ/veh, ^$$^*P* < 0.01, ^$$$^*P* < 0.001, ^$$$$^*P* < 0.0001 vs Aβ/DPZ0.25. **(F)** Time spent in target quadrant during the probe test (day 16). One-sample *t*-test: *P < 0.05 and **P < 0.01 vs chance level. **(B–F)** Bars in black indicate combination treatments. Data are expressed as mean ± SEM, *n* = 10 mice per group.

Mice were i.c.v., injected with Aβ_25__–__35_ peptide or Sc.Aβ on day 0 and chronically treated with DPZ and/or MEF from days 1 to 16 (Figure 2A). Familiarization phase demonstrated that these treatments had no effect on the time spent in exploring identical objects (all, *P* > 0.05; Figure 2B). In contrast during the NOR test phase, Aβ administration abolished the increase in exploration time of the novel object (*P* = 0.25; Figure 2C). While MEF1, DPZ0.25, or the combination DPZ0.25 + MEF0.1 did not reverse the Aβ-induced impairment, mice treated with DPZ0.25 + MEF0.3 or DPZ0.25 + MEF1 demonstrated a marked improvement in preference index (*P* = 0.021 and *P* = 0.0017, respectively) up to that obtained with DPZ at 1 mg/kg.

From days 11 to 16, mice were challenged with the MWM test. Amyloid β i.c.v., injection induced a significant memory deficit (from learning days 3–5) in comparison with Sc.Aβ injection (for all days, *P* < 0.0001; Figure 2D). DPZ0.25 or MEF1 showed no effect, whereas DPZ1 significantly reversed the Aβ-induced memory deficits from learning days 2 to 5 (*P* < 0.001 and *P* < 0.0001 for day 2 and days 3–5, respectively; vs Aβ/VEH; Figure 2D). In addition, DPZ0.25 combined with MEF0.3 or MEF1 showed a significantly superior learning memory activity (from learning days 2–5) when compared to DPZ0.25 alone.

On experimental day 16 (probe phase), the time spent in each of the four quadrants of the circular pool was measured during a 60-s session (Figure 2F). Interestingly, data highlighted the significantly superior cognitive activity of DPZ1 and DPZ0.25 + MEF1 groups (*P* = 0.013 and *P* = 0.0063. respectively; Figure 2F) as those treatments restored performance up to the level noted in Sc.Aβ-injected control mice (*P* = 0.011).

### Mefloquine Increased DPZ-Induced ACh Overflow in the Hippocampus but Did Not Alter DPZ-Inhibition of AChE Activity

**FIGURE 3:**
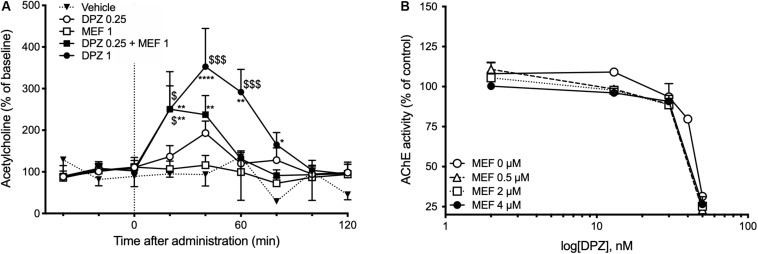
Effects of mefloquine (MEF) on donepezil-induced acetylcholine outflow **(A)** or donepezil (DPZ)-induced inhibition of acetylcholinesterase (AChE) activity **(B)**. **(A)** Acetylcholine (ACh) levels in 20-min collected microdialysate samples are expressed as percentages of the basal level calculated as the mean value of the three samples collected prior to administrations of MEF (1 mg/kg, i.p.), DPZ (0.25; 1 mg/kg, i.p.), or vehicle (NaCl 0.9%). Values are expressed as means ± SEM, *n* = 4–7 per group. Two-way ANOVA followed by Fisher least significant difference: *P < 0.05, **P < 0.01 vs Vehicle; ^$^*P* < 0.05, ^$$$^*P* < 0.001 vs DPZ0.25. **(B)** Concentration-dependent inhibition by DPZ (0, 2, 13, 30, 40, and 50 nM), in the absence or presence of MEF (0.5–4 μM), of mouse hippocampus AChE activity. Acetylcholinesterase activity is expressed as percent of that determined in the absence of drugs. Each data point represents the mean ± SEM of triplicate determinations in two independent experiments.

The effects of DPZ alone or combined with MEF on ACh outflow in the hippocampus were assessed using intracerebral microdialysis in awake freely moving mice. Intraperitoneal administration of DPZ1 alone or DPZ0.25 in combination with MEF1 caused a transient ACh overflow (*P* < 0.01, Figure 3A). Twenty minutes after injection, hippocampal ACh levels were increased by up to 2.5-fold in DPZ1 and DPZ0.25 + MEF1 groups and were significantly higher than in mice treated with the vehicle (both *P* < 0.01, Figure 3A) or DPZ0.25 alone (both, *P* < 0.05, Figure 3A). Administration of DPZ0.25 or MEF1 alone did not significantly modify ACh outflow.

*In vitro* assays were used to quantify the inhibitory effect of DPZ alone or combined with MEF on AChE activity in brain extracts. The addition of MEF (0, 0.5, 2, and 4 μM) did not modify the potency of DPZ to inhibit AChE activity (Figure 3B).

### Mefloquine Did Not Affect Brain and Serum Accumulation of DPZ

**FIGURE 4:**
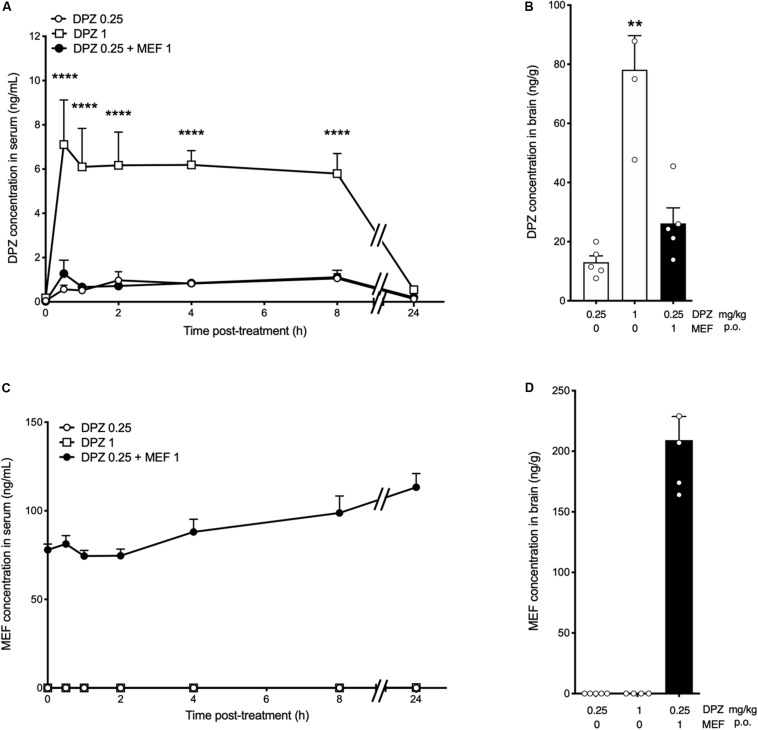
Serum and brain levels of donepezil and mefloquine after chronic treatments in rats. **(A–D)** Donepezil (DPZ, 0.25, 1 mg/kg) alone or in combination with mefloquine (MEF, 1 mg/kg) was administered p.o. daily for 14 days. **(A,C)** Blood samples were collected before treatment (T_0_) and then at 0.5, 1, 2, 4, 8, and 24 h after the last administration on day 14. **(B,D)** Brain samples were collected on day 15, 24 h after the last drug administration. Donepezil and MEF concentrations in rat serum [ng/mL, **(A,C)**] and brain [ng/g, **(B,D)**] are the means ± SEM of *n* = 4 to 10 animals per group. Two-way ANOVA followed by Bonferroni *post hoc* test, ****P < 0.0001 vs DPZ0.25 **(A)**. Kruskal–Wallis followed by Dunn *post hoc* test: **P < 0.01 vs DPZ0.25 **(B)**. No statistical analysis applicable **(C,D)**. Bars in black indicate combination treatments.

Donepezil (0.25 or 1 mg/kg) alone or in combination with MEF (1 mg/kg) was administered p.o., daily for 14 days, and the drugs were quantified in serum using an LC-MS method. The daily coadministration of MEF1 did not change the serum concentration of DPZ determined after 14-day treatment with 0.25 mg/kg p.o., daily of the latter drug (*P* > 0.05; Figure 4A). Brain concentration of DPZ was dose-dependent (DPZ0.25: 13.00 ± 2.17 ng/g, DPZ1: 78.1 ± 11.5 ng/g, *P* = 0.0036; Figure 4B). No significant difference was found between the brain concentrations of DPZ comparing DPZ0.25 + MEF1 (26.20 ± 5.26 ng/g) and DPZ0.25 groups (*P* = 0.33; Figure 4B). Mefloquine concentration in serum ranged between 78 and 113 ng/mL all along the 24 h after the last drug administration on the 14th treatment day (Figure 4C). In brain, MEF concentration reached 229.0 ± 19.5 ng/g 24 h after the last drug administration (Figure 4D).

### Inhibition of Cx30 or Cx43 in Hippocampal Astrocytes Improved the Capacity of DPZ to Prevent Scopolamine-Induced Memory Deficit

**FIGURE 5:**
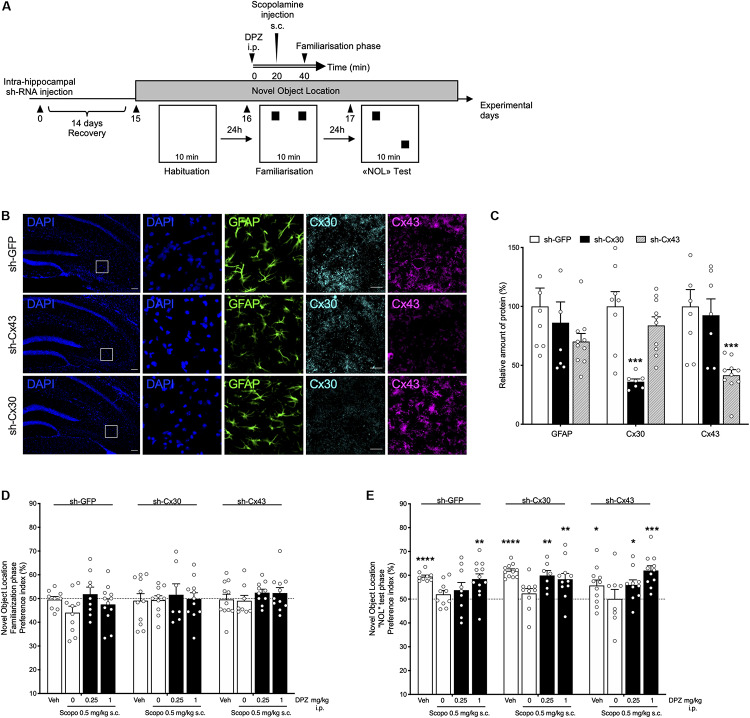
Behavioral effects of donepezil (DPZ) on scopolamine (Scopo)–induced deficits in mice with down-regulated hippocampal shCx43 or shCx30. **(A)** Experimental design: novel object location task. Recombinant lentiviruses aimed at locally down-regulating either Cx30 (sh-Cx30) or Cx43 (sh-Cx43) expression or that of GFP (sh-GFP, as control) specifically in astrocytes were administered into the hippocampus, on both sides. Two weeks later, DPZ (0.25, 1 mg/kg, i.p.) or vehicle (veh, DMSO 2%, NaCl 0.9%, i.p.) was administered 40 min before the familiarization phase (day 16) of the task. Scopolamine (0.5 mg/kg, s.c.) was administered 20 min later, that is, 20 min before the familiarization phase. [**(B)**, from left to right] Representative fluorescence microscopy images of hippocampal nuclei (DAPI, blue), GAFP (green), Cx30 (light blue), and Cx43 (purple) labeling (square boxes on DAPI-labeled sections correspond to the four series of images on the right). Scale bar: 100 μm. **(C)** Relative hippocampal expression of GFAP, Cx43, or Cx30 in sh- GFP-, sh-Cx43- and sh-Cx30-injected mice. Data are expressed as mean ± SEM, *n* = 7 to 10 mice per group. Two-way ANOVA followed by Tukey *post hoc* test, ^∗∗∗^*P* < 0.001, significantly different from the corresponding group injected with sh-GFP. **(D,E)** Preference index for the similar objects **(D)** and the displaced object **(E)**. One-sample *t*-test: ^∗^*P* < 0.05;^**^P < 0.01; ^∗∗∗^*P* < 0.001; ^****^P < 0.0001 vs chance level. Data are expressed as mean ± SEM, *n* = 7 to 12 mice per group.

Lentiviral vectors expressing shRNA against Cx30 or Cx43 (or control) were bilaterally injected into the hippocampus. Mice were tested in the NOL task after a 14-day recovery period (Figure 5A).

DAPI labeling and GFAP immunolabeling showed that the hippocampal density of all cell types, and especially astrocytes, did not differ between the groups of LV-injected mice (Figures 5B,C). Quantification of brain sections confirmed that Cx30 and Cx43 hippocampal expression was significantly decreased in sh-Cx30– and sh-Cx43–injected mice (*P* = 0.0007), respectively, compared to sh-GFP–injected group (Figure 5C).

During the familiarization phase of the NOL task, LV-injected mice examined the two identical objects for a similar exploration time (all groups, *P* > 0.05 vs chance level; Figure 5D). The duration of exploration of the displaced object was similarly enhanced in each LV-injected group given vehicle (*P* < 0.0001 for sh-GFP and sh-Cx30; *P* = 0.034 for sh-Cx43; Figure 5E). In contrast, all the LV-injected mice treated with scopolamine presented a preference index close to 50% showing clear-cut memory impairment. In all groups, a DPZ1 was able to prevent NOL memory deficits induced by scopolamine (*P* = 0.0027 for sh-GFP; *P* = 0.0066 for sh-Cx30 and *P* = 0.0001 for sh-Cx43 mice; Figure 5E). Interestingly, although DPZ0.25 was unable to prevent the memory deficit caused by scopolamine in sh-GFP injected mice (*P* = 0.28), this dose significantly reduced the scopolamine effect in both sh-Cx30 and sh-Cx43 groups (*P* = 0.0033 and *P* = 0.019, respectively; Figure 5E).

## Discussion

Astroglial Cxs play a central role in physiological functions and notably in neuronal modulations (Han et al., 2014; Dallerac and Rouach, 2016; Clasadonte et al., 2017). Previous studies proposed that astroglial networks may contribute to strengthen neuronal signaling in CNS disorders (Duchêne et al., 2016; Jeanson et al., 2016; Charvériat et al., 2017; Vodovar et al., 2018; Sauvet et al., 2019). Interestingly, expression of both Cx43 and Cx30 was found to be upregulated in AD mice models (Mei et al., 2010) and brains from AD patients (Nagy et al., 1996), supporting the idea that Cxs may also play a role in neuronal dysfunction in AD (Orellana et al., 2009; Giaume et al., 2017). It has also been reported that intense Cx blockade alleviated memory impairments in an AD mouse model (Takeuchi et al., 2012). Taken together, these data led us to investigate how Cx modulations influence the procognitive action of DPZ. In our studies, pharmacological modulation of Cxs was achieved using MEF, a widely used and potent Cx blocker (Cruikshank et al., 2004; Picoli et al., 2012; Jeanson et al., 2016) able to cross the blood–brain barrier (Baudry et al., 1997). In addition, LV-induced down-regulation of hippocampal Cx was also used to evaluate Cx-modulation of DPZ procognitive effects.

Effects of DPZ alone or combined with MEF on memory and learning performances were quantified using two mouse AD models. Scopolamine-treated animals were evaluated using Y-maze test and object recognition tests as commonly used to evaluate immediate or long-term spatial working memory and episodic memory (de Bruin et al., 2010; Hamlin et al., 2013). As second chronic model, we used mice that had been infused with amyloid-β_25__–__35__)_ (i) producing a significant neuronal loss (Maurice et al., 1996) and (ii) exhibiting a marked deficits in long-term learning and memory in NOR and MWM tasks (Fang and Liu, 2006; Tsunekawa et al., 2008). Our data showed that neither MEF nor DPZ at a low dose was able to reverse the memory deficits in those models but that the procognitive effect resulting from their combination fully compensated for these impairments, as efficiently as a high dose of DPZ.

We first investigated potential pharmacokinetic interactions between both drugs. Our data showed that concomitant MEF administration did not interfere with serum or brain accumulation of DPZ after a 2-week treatment. On the other hand, we found that MEF at low concentration (<4 μM) failed to affect DPZ-induced inhibition of AChE activity in brain extracts. Interestingly, under our conditions, MEF was devoid of any effect on AChE activity, and even at 10 times the concentration reached in the brain, in agreement with previous studies (Mcardle et al., 2005). Accordingly, the promoting effect of MEF on DPZ procognitive actions could not be ascribed to some additive inhibitory effects on AChE activity as brain concentration of MEF after 1 mg/kg treatment remains below micromolar range [Baudry et al. (1997) and in the present study]. Therefore, it can be proposed that the enhancing effect of MEF on DPZ-induced ACh overflow could not be underlain through pharmacokinetic mechanisms or direct effect on the AChE enzyme.

In addition to inhibiting AChE, DPZ also activates sigma-1 receptor (Maurice et al., 2006). However, at a concentration as high as 1 μM, MEF does not interfere with sigma-1 receptor (Eurofins CEREP test, data not shown), which makes very unlikely any potential implication of this receptor in the effects of THN201 vs DPZ.

On the other hand, MEF has been reported to have low affinity for various serotonin receptors notably 5-HT_1__A_, 5-HT_2__C_, and 5-HT_3_ receptors (Thompson et al., 2007; Janowsky et al., 2014) and noradrenaline transporters (Janowsky et al., 2014). However, MEF affinity for these monoaminergic targets was probably too low, with Ki values always greater than 1 μM, to allow any significant participation of them in mechanisms underlying MEF-induced potentiation of DPZ action. Indeed, under our *in vivo* treatment conditions, the brain concentration of MEF hardly reached 0.5 μM.

In sharp contrast with that noted about these aforementioned targets, MEF efficiently reduces Cx-mediated astroglial cellular coupling at the same concentration range as that reached under our treatment conditions (Jeanson et al., 2016). Furthermore, previous studies reported that global deletion of Cx43 (Frisch et al., 2003) and Cx30 (Dere et al., 2003) in astrocytes affected exploration, emotionality, and behavior; additionally, DPZ was previously shown not to significantly alter astrocyte Cx expression levels by itself (Biswas et al., 2018). This led us to examine the possible role of these gap junction proteins in the enhancement by MEF of the procognitive action of DPZ. To this goal, we selected a regional and incomplete inhibition of astroglial Cx expression in the hippocampus, a region concerned in the action of DPZ (Okamura et al., 2008). Accordingly, Cx30 or Cx43 expression was down-regulated specifically in hippocampal astrocytes using a pseudo-typed recombinant LV (Colin et al., 2009; Quesseveur et al., 2015). We thus showed that selective inhibition of astroglial Cx30 or Cx43 expression in the hippocampus did not induce any memory impairment and did not interfere with scopolamine-induced cognitive deficits in the object location task in mice. However, this selective inhibition was found to promote the capacity of DPZ at a low dose to counteract scopolamine-induced cognitive deficits in mice. These data closely resembled those found previously with the Cx inhibitor MEF, supporting the idea that astroglial Cx43 and Cx30 constitute new non-neuronal key proteins implicated in the pharmacological action of DPZ.

## Conclusion

Our study is the first addressing the role of astroglial Cxs as new therapeutic targets to enhance DPZ pharmacodynamic actions. We demonstrated that the Cx inhibitor MEF at a low dose enhanced the procognitive action of DPZ in mouse models ofAD. In addition, we provided clear-cut evidence that astroglial Cx30 and Cx43 exerted a regulatory influence on the capacity of DPZ to counteract scopolamine-induced memory deficits. Our present data with THN201 open promising perspectives toward improved treatments of cognitive impairments in AD patients.

## Data Availability Statement

All datasets presented in this study are included in the article/supplementary material.

## Ethics Statement

All experiments were conducted in strict conformity with the Policies of the French Ethics Committee. Animal surgery and experimentations conducted in this study were authorized by the French Direction of Veterinary Services (APAFIS#12311-2017071816217821 v3).

## Author Contributions

AD, MD, JM, and VV performed the behavioral experiments. BP, CL, and BG performed the LV-injections and micro dialysis. BP and MD analyzed the data. AD, MD, BG, and MC designed the experiments and managed the project. ND supervised the production of the viral vectors. MD and MC wrote the first draft. MD, AD, CP, BP, BG, MH, FM, and MC reviewed the manuscript. All authors approved the final manuscript.

## Conflict of Interest

JM and VV are full-time employees of Amylgen. MD, AD, CP, FM, and MC are full-time employees of Theranexus company. MH is a consultant for Theranexus company. The authors declare that this study received funding from Theranexus. The funder was involved in: the study design; collection, analysis, and interpretation of data; writing of the report. The authors had no restrictions regarding the submission of the report for publication. The authors had full access to the data, this access is on-going. The remaining authors (BP, CL, BG, and ND) declare that the research was conducted in the absence of any commercial or financial relationships that could be construed as a potential conflict of interest.
